# Anti-seizure Medication Prescription in Adult Outpatients With Epilepsy in China, 2013–2018

**DOI:** 10.3389/fneur.2021.649589

**Published:** 2021-05-24

**Authors:** Lingyan Yu, Wenjie Zhu, Xiuping Zhu, Yan Lu, Zhenwei Yu, Haibin Dai

**Affiliations:** ^1^Department of Pharmacy, Second Affiliated Hospital, Zhejiang University School of Medicine, Hangzhou, China; ^2^Department of Pharmacy, Sir Run Run Shaw Hospital, Zhejiang University School of Medicine, Hangzhou, China

**Keywords:** anti-seizure medications, prescription, China, levetiracetam, sodium valproate, epilepsy

## Abstract

This study aimed to assess the national trends in anti-seizure medication (ASM) prescription in Chinese adult outpatients with epilepsy over a 6-year period from 2013 to 2018. Prescriptions for adult outpatients with epilepsy from hospitals in six major cities were extracted from the database of the Hospital Prescription Analysis Cooperative Project. Trends in the annual prescriptions and expenditure of ASM were analyzed. Prescription patterns (monotherapy or combination therapy) were also assessed. A total of 225,767 prescriptions from 60 hospitals were eligible and extracted for analysis. The number of ASM prescriptions increased from 28,360 in 2013 to 44,110 in 2018, and the corresponding cost increased from 9,452,990 Chinese Yuan (CNY) in 2013 to 14,627,865 CNY in 2018. The share of newer ASM use increased continuously, accounting for 56.75% of prescriptions and 85.03% of expenditure in 2018. The most frequently prescribed ASMs were sodium valproate and levetiracetam. The proportion of sodium valproate use decreased, while the proportion of levetiracetam use increased dramatically in terms of both ASM prescriptions and expenditure. Monotherapy was more frequent than combination therapy. The three most common combination therapies were sodium valproate/lamotrigine, levetiracetam/oxcarbazepine, and sodium valproate/levetiracetam. In summary, ASM use increased rapidly in terms of the number of ASM prescriptions and cost during the 6-year period, which raises concern regarding the rational use and pharma-economic profiles of ASMs. In place of valproate, levetiracetam became the most frequently used ASM. The development of ASM prescription is in line with therapy guidelines and reflects the current state of research in China.

## Introduction

Epilepsy is the third leading contributor to the global burdens of disability, mortality, morbidity, stigma, and costs related to neurological disorders (1). A meta-analysis reported that the annual global cumulative incidence of epilepsy was 67.77 per 100,000 persons (2). Up to 70% of people with epilepsy could become seizure free if appropriately diagnosed and treated (3). Therefore, better treatment and management of patients with epilepsy are urgently needed. For now, anti-seizure medication (ASM) remains the main choice for patients with epilepsy, who sometimes require lifelong treatment (4). ASMs exert pharmacological action in the treatment of epileptic disorders, aiming to achieve seizure freedom or at least to improve seizure control. The choice of ASM is influenced by many factors, such as seizure type, epilepsy syndrome, other medications used, patient age, current guidelines, cost, and the prescribing doctor's experience (5). Thus, understanding the current situation regarding ASM usage is critical, as it could improve epilepsy management.

In recent years, a number of studies have investigated ASM prescription patterns in various countries and found various trends (6–8). We also previously described the trends in ASM use in pediatric patients in China (9). However, ASM use in adult Chinese patients remains unclear. The aim of this study was to evaluate the trends and patterns in ASM use over a 6-year period using prescription data from six major cities.

## Methods

### Study Design

This study was designed as a cross-sectional study based on prescription data. Ethical approval was obtained from the ethics committee of the Second Affiliated Hospital of Zhejiang University, School of Medicine (reference number 2020-719). Informed consent was waived as part of the approval.

### Data Source and Study Sample

The prescriptions were extracted from the database of the Hospital Prescription Analysis Cooperative Project, which was used in our previous studies and many other Chinese epidemiological studies (9–13). The database contains prescription information from participating hospitals on 40 random days each year.

Prescriptions that met the following criteria were included: (1) issued for outpatients aged >18 years; (2) issued for patients with a diagnosis of epilepsy (regardless of diagnostic criteria, type, or severity); (3) containing at least one ASM (initial prescription or a renewal) and issued between 2013 and 2018; and (4) from hospitals in Beijing, Shanghai, Hangzhou, Chengdu, Guangzhou, or Tianjin that participated in the program continuously from 2013 to 2018. The following items were extracted from the included prescriptions: prescription code, sex and age of patient, year issued, location, hospital code, diagnosis, generic drug names, and cost of each drug.

### Analysis

The primary analysis units were ASM prescriptions (outpatient prescriptions, initial prescription, or renewal) and expenditure. The total expenditure was calculated by summing all the costs of ASM prescriptions. The overall trends in ASM use were described using the estimated annual number of ASM prescriptions and annual expenditure (based on the available data from 40 random days each year). The trends were further stratified by age, sex, ASM class, and specific ASM.

ASMs were classified as older and newer agents, as in our previous study on ASM use in children (9, 14). Prescription patterns were categorized into monotherapy (treatment with one ASM) and polytherapy (concurrent treatment with two or more ASMs).

The statistical significance of the trends in proportions was assessed by log-linear analysis. Other trends were analyzed using the Mann–Kendall trend test. The statistical analysis was conducted in R (3.5.0).

## Results

### Overall Trends in Anti-seizure Medication Use

**Figure 1:**
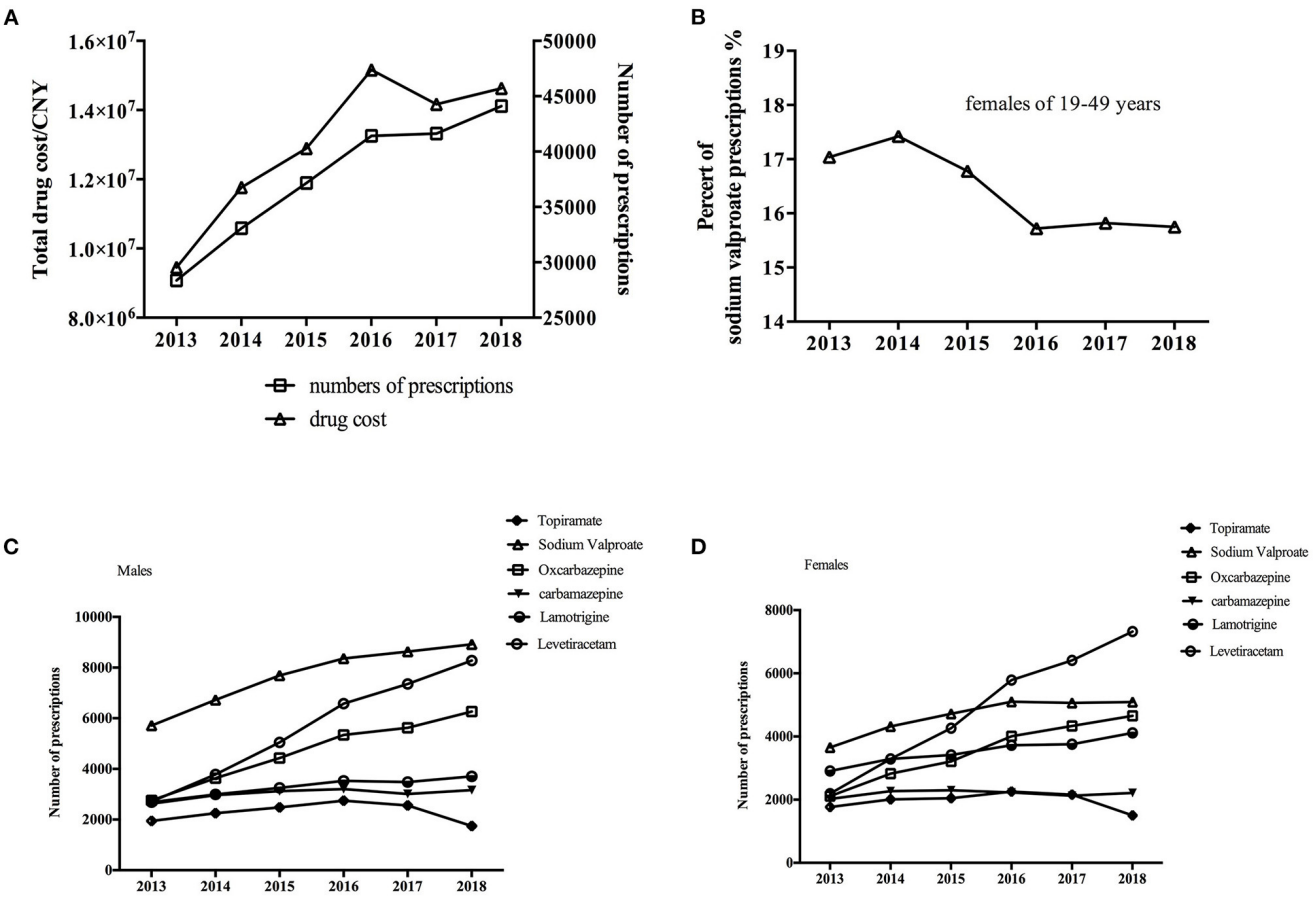
**(A)** Trends in overall prescriptions and cost of anti-seizure medications (ASM) from 2013 to 2018. **(B)** Trends in sodium valproate use in females of child-bearing age (19–49 years old). Trends in individual ASM use in **(C)** males and **(D)** females from 2013 to 2018.

A total of 225,767 prescriptions from 60 hospitals met the inclusion criteria and were included. The number of ASM prescriptions continuously increased from 28,360 in 2013 to 44,110 in 2018 (Figure 1A, *P* < 0.05). The corresponding expenditure also increased, from 9,452,990 Chinese Yuan (CNY) in 2013 to 14,627,865 CNY in 2018 (one CNY was about 0.15 US dollar or 0.13 Euro; Figure 1A, *P* < 0.05).

ASM use by sex and age is shown in Table 1. The prevalence of ASM use was higher in males than in females, and the ratio of males to females remained constant over the 6-year period (*P* > 0.05). The most common age groups regarding ASM use were 19–30 and 51–60 years. The proportion of prescriptions for patients aged 19–30 continuously decreased (*P* < 0.05), while that for patients aged >60 increased dramatically (*P* < 0.05).

**Table 1:** Demographic characteristics of study sample from 2013 to 2018.

	**Number of patients (%)**								
	**2013**	**2014**	**2015**	**2016**	**2017**	**2018**	**Total**	***P*_**1**_**	***P*_**2**_**
**Age (years)**									
19–30	7,163 (25.26)	7,892 (23.85)	8,408 (22.63)	9,347 (22.56)	9,056 (21.76)	9,136 (20.71)	51,002 (22.59)	0.060	0.001
31–40	4,451 (15.69)	5,503 (16.63)	6,190 (16.66)	6,679 (16.12)	7,102 (17.06)	7,872 (17.85)	37,797 (16.74)	0.009	0.044
41–50	4,410 (15.55)	4,955 (14.98)	5,465 (14.71)	5,924 (14.30)	6,084 (14.62)	6,388 (14.48)	33,226 (14.72)	0.009	0.045
51–60	5,542 (19.54)	6,666 (20.15)	7,408 (19.93)	8,175 (19.73)	7,564 (18.17)	7,659 (17.36)	43,014 (19.05)	0.060	0.048
61–70	3,544 (12.50)	4,369 (13.21)	5,498 (14.79)	6,764 (16.33)	7,062 (16.97)	7,849 (17.79)	35,086 (15.54)	0.009	<0.001
>70	3,250 (11.46)	3,700 (11.18)	4,193 (11.28)	4,541 (10.96)	4,752 (11.42)	5,206 (11.80)	25,642 (11.36)	0.009	0.450
**Sex**									
Male	15,774 (55.62)	18,317 (55.36)	20,870 (56.16)	23,032 (55.59)	23,081 (55.46)	24,548 (55.65)	125,622 (55.64)	0.009	0.963
Female	12,586 (44.38)	14,768 (44.64)	16,292 (43.84)	18,398 (44.41)	18,539 (44.54)	19,562 (44.35)	100,145 (44.36)	0.009	0.961

*P_1_, P-value for trend in number of prescriptions, assessed by Mann–Kendall trend test; P_2_, P-value for trend in proportion of prescriptions, assessed by log-linear analysis*.

### Trends in Anti-seizure Medication Use by Anti-seizure Medication Class and Individual Anti-seizure Medication

The annual number of prescriptions and expenditure by ASM class and individual ASM is shown in Tables 2, 3. The proportion of older ASM use significantly decreased from 48.75% in 2013 to 38.67% in 2018 (*P* < 0.05). Meanwhile, the expenditure on older ASMs significantly decreased from 16.84% in 2013 to 13.23% in 2018 (*P* < 0.05). At the end of the study, new ASMs accounted for 56.75% of prescriptions and 85.03% of the expenditure.

**Table 2:** Number of anti-seizure medication (ASM) prescriptions from 2013 to 2018.

**Medicine**	**2013**	**2014**	**2015**	**2016**	**2017**	**2018**	***P*_**1**_**	***P*_**2**_**
Older AEDs	18,291 (48.75)	20,947 (46.37)	22,736 (44.55)	23,821 (41.10)	23,987 (40.09)	25,020 (38.67)	0.009	<0.001
Carbamazepine	4,654 (12.40)	5,230 (11.58)	5,420 (10.62)	5,434 (9.38)	5,137 (8.59)	5,376 (8.31)	0.452	<0.001
Clonazepam	1,866 (4.97)	2,073 (4.59)	2,303 (4.51)	2,477 (4.27)	2,809 (4.69)	3,246 (5.02)	0.009	0.925
Phenobarbitone	929 (2.48)	1,033 (2.29)	991 (1.94)	1,003 (1.73)	960 (1.60)	886 (1.37)	0.452	<0.001
Phenytoin	806 (2.15)	789 (1.75)	809 (1.59)	752 (1.30)	741 (1.24)	746 (1.15)	0.133	0.001
Primidone	38 (0.10)	44 (0.10)	40 (0.08)	49 (0.08)	42 (0.07)	43 (0.07)	0.452	0.005
Sodium valproate	9,368 (24.97)	11,042 (24.44)	12,411 (24.32)	13,464 (23.23)	13,695 (22.89)	14,008 (21.65)	0.009	0.001
Magnesium valproate	343 (0.91)	449 (0.99)	475 (0.93)	503 (0.87)	449 (0.75)	523 (0.81)	0.009	0.055
Valpromide	287 (0.76)	287 (0.64)	287 (0.56)	139 (0.24)	154 (0.26)	192 (0.30)	0.314	0.023
Newer AEDs	19,227 (51.25)	24,224 (53.63)	28,304 (55.45)	34,139 (58.90)	35,848 (59.91)	39,680 (61.33)	0.009	<0.001
Gabapentin	144 (0.38)	147 (0.33)	151 (0.30)	173 (0.30)	170 (0.28)	176 (0.27)	0.024	0.010
Lamotrigine	5,594 (14.91)	6,284 (13.91)	6,664 (13.06)	7,250 (12.51)	7,239 (12.10)	7,823 (12.09)	0.024	0.002
Levetiracetam	4,901 (13.06)	7,068 (15.65)	9,311 (18.24)	12,366 (21.34)	13,764 (23.00)	15,602 (24.11)	0.009	0.001
Oxcarbazepine	4,874 (12.99)	6,462 (14.31)	7,645 (14.98)	9,350 (16.13)	9,957 (16.64)	10,921 (16.88)	0.009	0.001
Topiramate	3,712 (9.89)	4,261 (9.43)	4,530 (8.88)	5,000 (8.63)	4,718 (7.89)	5,158 (7.97)	0.024	0.001
Zonisamide	2 (0.01)	2 (0.00)	3 (0.01)	0 (0.00)	0 (0.00)	0 (0.00)	NA	NA

*P_1_, P-value for trend in number of prescriptions, assessed by Mann–Kendall trend test; P_2_, P-value for trend in proportion of prescriptions, assessed by log-linear analysis; NA, not tested; AED, antiepileptic drug*.

**Table 3:** Total expenditure on anti-seizure medication (ASM) dispensed from 2013 to 2018.

**Medicine**	**2013**	**2014**	**2015**	**2016**	**2017**	**2018**	***P*_**1**_**	***P*_**2**_**
Older AEDs	1,591,483 (16.84)	1,830,187 (15.55)	2,050,545 (15.90)	2,166,017 (14.28)	1,983,533 (14.00)	1,935,966 (13.23)	0.452	0.002
Carbamazepine	319,127 (3.38)	344,937 (2.93)	355,135 (2.75)	357,934 (2.36)	318,717 (2.25)	324,209 (2.22)	1.000	0.001
Clonazepam	18,584 (0.20)	20,490 (0.17)	24,455 (0.19)	29,791 (0.20)	39,069 (0.28)	51,282 (0.35)	0.009	0.033
Phenobarbitone	2,577 (0.03)	2,802 (0.02)	9,017 (0.07)	19,639 (0.13)	19,516 (0.14)	18,214 (0.12)	0.133	0.015
phenytoin	5,821 (0.06)	5,326 (0.05)	5,615 (0.04)	13,145 (0.09)	14,549 (0.10)	14,513 (0.10)	0.133	0.076
Primidone	1,220 (0.01)	1,580 (0.01)	3,678 (0.03)	6,389 (0.04)	4,967 (0.04)	5,655 (0.04)	0.060	0.021
Sodium valproate	1,169,808 (12.38)	1,359,643 (11.55)	1,557,194 (12.08)	1,648,508 (10.87)	1,509,352 (10.65)	1,440,354 (9.85)	0.452	0.007
Magnesium valproate	63,296 (0.67)	83,576 (0.71)	84,295 (0.65)	81,775 (0.54)	70,217 (0.50)	73,235 (0.50)	1.000	0.010
Valpromide	11,051 (0.12)	11,833 (0.10)	11,156 (0.09)	8,836 (0.06)	7,147 (0.05)	8,504 (0.06)	0.133	0.008
Newer AEDs	7,861,508 (83.16)	9,938,515 (84.45)	10,844,467 (84.10)	12,997,937 (85.72)	12,187,396 (86.00)	12,691,899 (86.77)	0.060	0.002
Gabapentin	16,867 (0.18)	13,966 (0.12)	14,964 (0.12)	15,525 (0.10)	14,756 (0.10)	13,359 (0.09)	0.260	0.018
Lamotrigine	1,964,445 (20.78)	1,934,179 (16.43)	1,880,111 (14.58)	2,002,574 (13.21)	1,816,131 (12.82)	1,775,650 (12.14)	0.133	0.004
Levetiracetam	2,903,556 (30.72)	4,367,476 (37.11)	5,117,049 (39.68)	6,551,751 (43.21)	6,468,536 (45.65)	6,965,374 (47.62)	0.024	0.002
Oxcarbazepine	2,064,614 (21.84)	2,613,656 (22.21)	2,798,978 (21.71)	3,356,287 (22.13)	3,014,267 (21.27)	3,058,623 (20.91)	0.060	0.087
Topiramate	911,587 (9.64)	1,008,142 (8.57)	1,031,171 (8.00)	1,071,800 (7.07)	873,705 (6.17)	878,893 (6.01)	1.000	<0.001
Zonisamide	439 (0.00)	1,097 (0.01)	2,193 (0.02)	0 (0.00)	0 (0.00)	0 (0.00)	NA	NA

*Expenditure is expressed in Chinese Yuan (CNY). One CNY was about 0.15 US dollar or 0.13 Euro*.

*P_1_, P-value for trend in expenditure, assessed by Mann–Kendall trend test; P_2_, P-value for trend in proportion of expenditure, assessed by log-linear analysis; NA, not tested*.

The most commonly prescribed ASM at the beginning of the study was sodium valproate. The number of sodium valproate prescriptions increased over the study period, but the proportion of sodium valproate prescriptions among ASM prescriptions decreased (both *P* < 0.05). Levetiracetam use concomitantly increased from 13.06% in 2013 to 24.11% in 2018 (*P* < 0.05), and it became the most commonly used ASM, associated with the highest expenditure, at the end of the study. Other commonly prescribed ASMs included oxcarbazepine and lamotrigine, with the proportion of oxcarbazepine use increasing and the proportion of lamotrigine use decreasing (both *P* < 0.05). The proportion of carbamazepine use diminished from 12.40% in 2013 to 8.31% in 2018 (*P* < 0.05).

We also analyzed the use of individual ASMs by sex (Figures 1C,D). There were similar trends in the use of various ASMs between males and females except for sodium valproate use, which stopped increasing among female patients from 2016. More specifically, among female patients of child-bearing age (19–49 years old), the proportion of sodium valproate use decreased to its lowest level in 2016 and then plateaued (Figure 1B). Levetiracetam and oxcarbazepine use rapidly increased in both males and females (*P* < 0.05).

### Prescription Patterns

A total of 68.19% of ASM prescriptions involved monotherapy while 31.81% involved polytherapy (two or more ASMs). The most frequently used combination for adult patients was sodium valproate/lamotrigine (6,364 prescriptions), followed by levetiracetam/oxcarbazepine (6,098 prescriptions) and sodium valproate/levetiracetam (5,949 prescriptions).

## Discussion

This study investigated the trends in ASM use in Chinese adult patients over a 6-year period using a large database. It was found that both the number of patients receiving ASMs and the total cost increased. The proportion of patients receiving older ASMs slightly decreased. Newer ASMs accounted for nearly 56.75% of ASM prescriptions in 2018, and this amounted to 85.03% in costs.

Similar to our previous finding in children with epilepsy, increasing ASM use was observed in Chinese adult patients. This may be attributable to several factors. First, the prevalence of epilepsy in adults in China has been reported to increase in recent years (15). Second, the demand for better management among epilepsy patients has also increased with the improvements in their economic status. Third, epilepsy can occur following a cerebral stroke, which exhibits a high incidence in older patients (16), and the population is rapidly aging in China (17). It should be noted that ASM prescriptions for patients aged >60 years increased dramatically during the study period. Our results suggest that more attention should be paid to the rational use and management of ASMs, especially in older patients.

The corresponding expenditure also increased during the 6-year period. This emphasized the importance of considering the pharmaco-economic profile of ASMs. The economic pressure for patients who are living in poverty should be noted. Moreover, regulatory agents and insurance companies need to take the increases into account.

The use of older and newer ASMs varies among countries (18–20). The proportion of older ASM use among adults (38.67%) was higher than that among children (11.2%). However, the use decreased over the study period to a much greater extent in adults. The consumption of newer ASMs increased continuously. Newer ASMs are reported to be no more efficacious than older ASMs, but some offer advantages in terms of fewer drug interactions and better tolerance (21); physicians seemed to prefer drugs that can be managed easier. However, the cost of newer ASMs is higher than that of older ASMs, which necessitates pharmaco-economic evaluations.

There were pronounced changes in the use of specific ASMs over time, with decreases in the proportion of sodium valproate and carbamazepine prescriptions and increases in the proportion of levetiracetam and oxcarbazepine prescriptions (Table 2). Levetiracetam and sodium valproate were the two most frequently used ASMs, which has also been observed in England drug utilization research (22).

The number of sodium valproate prescriptions increased; however, the proportion continuously decreased. It was the most frequently used ASM at the beginning of the study, while levetiracetam took its place in 2017. Valproate is a broad-spectrum ASM, most commonly used in adults with generalized-onset seizures or generalized epilepsy, except for women of child-bearing age (23). For individuals with generalized-onset seizures, first-line treatment with sodium valproate is significantly more effective than carbamazepine, topiramate, and phenobarbitone (24). However, warnings about valproate use in girls have been issued (25). We evaluated ASM use by sex and found that all ASMs showed similar trends in males and females, except for sodium valproate. Similar to ASM use in children, sodium valproate use in female adults stopped increasing from 2016, which was the main reason that levetiracetam became the most commonly used ASM (Figure 1D) (26). It was observed that Chinese physicians have clearly accepted up-to-date guidelines well and have applied them in clinical practice.

Levetiracetam is most commonly approved as an adjunctive treatment for partial-onset seizures with or without secondary generalization (27). It has a high therapeutic index, but there remains a need to pay attention to adverse effects, such as irritability, anxiety, and aggressiveness (28). Carbamazepine was among the earliest traditional medications licensed for treating epileptic seizures, but the proportion of carbamazepine prescriptions decreased during the study period. However, unlike in pediatric patients (0.61%), carbamazepine prescriptions at the end of the study still amounted to a high proportion (8.31%) of the prescriptions. The main limitations of carbamazepine are potential drug interactions and the risk of severe adverse reactions, such as Stevens–Johnson syndrome. Oxcarbazepine has a similar structure and efficacy but a better safety profile (though it can cause severe skin reactions) (29). Its use slightly increased during the 6 years. Notably, the extent of the increase in oxcarbazepine use was almost equivalent to the decrease in carbamazepine use, which indicates that there may have been a switch from carbamazepine to oxcarbazepine. Regardless, as oxcarbazepine carries the risk of severe skin reactions, testing for the high-risk HLA-B^*^1502 allele in patients with relevant ancestry (i.e., genetically at-risk populations) would be beneficial (30).

ASM monotherapy is recommended for initial epilepsy treatment, and polytherapy is only recommended when monotherapy is not effective, as it may increase the risks of poor adherence, drug interactions, and toxicity (31). The most frequently used combination for adult patients was sodium valproate/lamotrigine, similar to the finding of a multicenter cross-sectional study in China (32). There were differences between our results and the results of a study in Kazakhstan, such as their most common combination therapy being valproate/carbamazepine (19). Although the debate regarding the most effective combination therapies for epilepsy is ongoing, using two drugs with complementary mechanisms is an interesting approach due to potential synergic effects and reduced risk of additional side effects.

This study had several limitations. Specific patients could not be identified, so the association between history of ASM use and outcomes could not be evaluated. Type of epilepsy was unknown, though this may determine the selection of specific ASMs. Finally, the included hospitals were all located in major cities, which may have caused sampling bias.

## Conclusion

ASM use increased in terms of both the number of prescriptions and expenditure between 2013 and 2018. The increasing use of newer ASMs is putting huge economic pressure on the health-care system. Levetiracetam replaced valproate as the most frequently used ASM. The changes in ASM prescriptions are in line with therapy guidelines and reflect the current state of research in China. Moreover, physicians seem to prefer safe and easily managed ASMs.

## Data Availability Statement

The original contributions presented in the study are included in the article/supplementary material, further inquiries can be directed to the corresponding author/s.

## Ethics Statement

The studies involving human participants were reviewed and approved by ethic committee of Second Affiliated Hospital of Zhejiang University, School of Medicine. Written informed consent for participation was not required for this study in accordance with the national legislation and the institutional requirements.

## Author Contributions

HD and LY: conceptualization and writing—review and editing. WZ and XZ: data curation. LY, WZ, XZ, and ZY: formal analysis. YL and HD: funding acquisition. LY, HD, and ZY: investigation. HD: methodology and resources. YL and ZY: validation. LY: visualization. LY and WZ: writing—original draft. All authors contributed to the article and approved the submitted version.

## Conflict of Interest

The authors declare that the research was conducted in the absence of any commercial or financial relationships that could be construed as a potential conflict of interest.
